# IMI2-PainCare-BioPain-RCT2 protocol: a randomized, double-blind, placebo-controlled, crossover, multicenter trial in healthy subjects to investigate the effects of lacosamide, pregabalin, and tapentadol on biomarkers of pain processing observed by non-invasive neurophysiological measurements of human spinal cord and brainstem activity

**DOI:** 10.1186/s13063-022-06431-5

**Published:** 2022-09-05

**Authors:** Caterina Leone, Giulia Di Stefano, Giuseppe Di Pietro, Petra Bloms-Funke, Irmgard Boesl, Ombretta Caspani, Sonya C. Chapman, Nanna Brix Finnerup, Luis Garcia-Larrea, Tom Li, Marcus Goetz, André Mouraux, Bernhard Pelz, Esther Pogatzki-Zahn, Andreas Schilder, Erik Schnetter, Karin Schubart, Irene Tracey, Inaki F. Troconiz, Hans Van Niel, Jose Miguel Vela Hernandez, Katy Vincent, Jan Vollert, Vishvarani Wanigasekera, Matthias Wittayer, Keith G. Phillips, Andrea Truini, Rolf-Detlef Treede

**Affiliations:** 1 Department of Human Neuroscience, Sapienza University, Rome, Italy; 2 Translational Science & Intelligence, Grünenthal GmbH, Aachen, Germany; 3 Clinical Science Development, Grünenthal GmbH, Aachen, Germany; 4 Department of Neurophysiology, Mannheim Center for Translational Neurosciences (MCTN), Medical Faculty Mannheim, University of Heidelberg, Mannheim, Germany; 5 Neuroscience Next Generation Therapeutics, Eli Lilly and Company, Lilly Innovation Center, Cambridge, MA USA; 6 Eli Lilly and Company, Arlington Square, Bracknell, UK; 7 Danish Pain Research Center, Department of Clinical Medicine, Aarhus University, Aarhus, Denmark; 8 Lyon Neurosciences Center Research Unit Inserm U 1028, Pierre Wertheimer Hospital, Hospices Civils de Lyon, Lyon 1 University, Lyon, France; 9 Teva Pharmaceutical Industries Ltd., Petah Tikva, Israel; 10 MRC Systems GmbH, Heidelberg, Germany; 11 Institute of Neuroscience (IoNS), UCLouvain, Brussels, Belgium; 12 Department of Anaesthesiology, Intensive Care and Pain Medicine, University Hospital Münster, Münster, Germany; 13 University Computing Centre, University of Heidelberg, Heidelberg, Germany; 14 ConsulTech GmbH, Berlin, Germany; 15 Wellcome Centre for Integrative Neuroimaging, Nuffield Department of Clinical Neurosciences, University of Oxford, Oxford, UK; 16 Department of Pharmaceutical Technology and Chemistry, School of Pharmacy and Nutrition, University of Navarra, Pamplona, Spain; 17 Mature Products Development, Grünenthal GmbH, Aachen, Germany; 18 Welab Barcelona, Barcelona, Spain; 19 Drug Discovery & Preclinical Development, ESTEVE Pharmaceuticals, Barcelona, Spain; 20 Nuffield Department of Women’s and Reproductive Health (NDWRH), University of Oxford, Oxford, UK; 21 Pain Research, Department of Surgery and Cancer, Imperial College London, London, UK

**Keywords:** Pain, Analgesics, PK/PD, Spinal cord, Biomarkers, RIII flexion reflex, N13 somatosensory evoked potentials, Hyperalgesia, RCT, Healthy subjects

## Abstract

**Background:**

IMI2-PainCare-BioPain-RCT2 is one of four similarly designed clinical studies aiming at profiling a set of functional biomarkers of drug effects on specific compartments of the nociceptive system that could serve to accelerate the future development of analgesics. IMI2-PainCare-BioPain-RCT2 will focus on human spinal cord and brainstem activity using biomarkers derived from non-invasive neurophysiological measurements.

**Methods:**

This is a multisite, single-dose, double-blind, randomized, placebo-controlled, 4-period, 4-way crossover, pharmacodynamic (PD) and pharmacokinetic (PK) study in healthy subjects. Neurophysiological biomarkers of spinal and brainstem activity (the RIII flexion reflex, the N13 component of somatosensory evoked potentials (SEP) and the R2 component of the blink reflex) will be recorded before and at three distinct time points after administration of three medications known to act on the nociceptive system (lacosamide, pregabalin, tapentadol), and placebo, given as a single oral dose in separate study periods. Medication effects on neurophysiological measures will be assessed in a clinically relevant hyperalgesic condition (high-frequency electrical stimulation of the skin), and in a non-sensitized normal condition. Patient-reported outcome measures (pain ratings and predictive psychological traits) will also be collected; and blood samples will be taken for pharmacokinetic modelling. A sequentially rejective multiple testing approach will be used with overall alpha error of the primary analysis split between the two primary endpoints, namely the percentage amplitude changes of the RIII area and N13 amplitude under tapentadol. Remaining treatment arm effects on RIII, N13 and R2 recovery cycle are key secondary confirmatory analyses. Complex statistical analyses and PK-PD modelling are exploratory.

**Discussion:**

The RIII component of the flexion reflex is a pure nociceptive spinal reflex widely used for investigating pain processing at the spinal level. It is sensitive to different experimental pain models and to the antinociceptive activity of drugs. The N13 is mediated by large myelinated non-nociceptive fibers and reflects segmental postsynaptic response of wide dynamic range dorsal horn neurons at the level of cervical spinal cord, and it could be therefore sensitive to the action of drugs specifically targeting the dorsal horn. The R2 reflex is mediated by large myelinated non-nociceptive fibers, its circuit consists of a polysynaptic chain lying in the reticular formation of the pons and medulla. The recovery cycle of R2 is widely used for assessing brainstem excitability. For these reasons, IMI2-PainCare-BioPain-RCT2 hypothesizes that spinal and brainstem neurophysiological measures can serve as biomarkers of target engagement of analgesic drugs for future Phase 1 clinical trials. Phase 2 and 3 clinical trials could also benefit from these tools for patient stratification.

**Trial registration:**

This trial was registered on 02 February 2019 in EudraCT (2019-000755-14).

## Administrative information

Note: the numbers in curly brackets in this protocol refer to SPIRIT checklist item numbers. The order of the items has been modified to group similar items (see http://www.equator-network.org/reporting-guidelines/spirit-2013-statement-defining-standard-protocol-items-for-clinical-trials/).

**Table:** 

Title {1}	IMI2-PainCare-BioPain-RCT2: A randomized, double-blind, placebo-controlled, cross-over, multicenter trial in healthy subjects to investigate the effects of lacosamide, pregabalin and tapentadol on biomarkers of pain processing observed by non-invasive neurophysiological measurements of human spinal cord and brainstem activity
Trial registration {2a and 2b}.	EudraCT registration: 2019-000755-14
Protocol version {3}	4.0 (12/06/2019)
Funding {4}	This project has received funding from the Innovative Medicines Initiative 2 Joint undertaking under grant agreement No 777500. This Joint Undertaking receives support from the European Union’s Horizon 2020 research and innovation program and EFPIA.
Author details {5a}	Caterina Leone*c1, Giulia Di Stefano*1, Giuseppe Di Pietro1, Petra Bloms-Funke2, Irmgard Boesl3, Ombretta Caspani4, Sonya C Chapman5, Nanna Brix Finnerup6, Luis Garcia-Larrea7, Tom Li8, Marcus Goetz9, André Mouraux10, Bernhard Pelz9, Esther Pogatzki-Zahn11, Andreas Schilder4, Erik Schnetter12, Karin Schubart13, Irene Tracey14, Inaki F. Troconiz15, Hans Van Niel16, Jose Miguel Vela Hernandez17, Katy Vincent18, Jan Vollert4,11,19, Vishvarani Wanigasekera14, Matthias Wittayer4, , Keith G Phillips**5, Andrea Truini**1, Rolf-Detlef Treede**4 1. Department of Human Neuroscience, Sapienza University, Rome, Italy. 2. Translational Science & Intelligence, Grünenthal GmbH, Aachen, Germany. 3. Clinical Science Development, Grünenthal GmbH, Aachen, Germany. 4. Department of Neurophysiology, Mannheim Center for Translational Neurosciences (MCTN), Medical Faculty Mannheim, University of Heidelberg, Mannheim, Germany. 5. Neuroscience Next Generation Therapeutics, Eli Lilly and Company, Lilly Innovation Center, Cambridge, MA, USA 6. Danish Pain Research Center, Department of Clinical Medicine, Aarhus University, Aarhus, Denmark. 7. Lyon Neurosciences Center Research Unit Inserm U 1028, Pierre Wertheimer Hospital, Hospices Civils de Lyon, Lyon 1 University, Lyon, France. 8. Teva Pharmaceutical Industries Ltd., Israel 9. MRC Systems GmbH, Heidelberg, Germany 10. Institute of Neuroscience (IoNS), UCLouvain, Brussels, Belgium. 11. Department of Anaesthesiology, Intensive Care and Pain Medicine, University Hospital Münster, Münster, Germany. 12. University Computing Centre, University of Heidelberg, Heidelberg, Germany. 13. ConsulTech GmbH, Berlin, Germany 14. Wellcome Centre for Integrative Neuroimaging, Nuffield Department of Clinical Neurosciences, University of Oxford, Oxford, United Kingdom 15. Department of Pharmaceutical Technology and Chemistry, School of Pharmacy and Nutrition, University of Navarra, Pamplona, Spain. 16. Mature Products Development, Grünenthal GmbH, Aachen, Germany. 17. Welab Barcelona, Barcelona, Spain 18. Nuffield Department of Women's and Reproductive Health (NDWRH), University of Oxford, Oxford, UK. 19. Pain Research, Department of Surgery and Cancer, Imperial College London, London, UK.
Name and contact information for the trial sponsor {5b}	Andrea Truini, Department of Human Neuroscience, Sapienza University of Rome, Viale Università 30, Rome, Italy. Telephone +390649914758
Role of sponsor {5c}	This study is one of four studies conducted in subtopic BioPain of the IMI2-PainCare project, coordinated by Rolf-Detlef Treede (Heidelberg University). Design of the study was led by the sponsor and coordinator, and involved all partners of the BioPain subtopic of the IMI2-PainCare consortium (WP5, WP6, WP7). The sponsor will coordinate data collection at all sites and extract all biomarker parameters from the source data. Final analysis of study endpoints will be coordinated by Rolf-Detlef Treede and involve all partners of the BioPain subtopic of the IMI2-PainCare consortium.

## Introduction

### Background and rationale {6a}

Chronic pain is one of the leading causes of human suffering and also a major social burden [1]. Currently available pharmacological therapies provide inadequate relief for many patients with chronic pain. Novel drugs which are efficacious analgesics in preclinical models often have little or no clinical efficacy, but it is often not known whether the drug engaged the human target sufficiently to have a meaningful pharmacodynamic effect. Hence, early deselection of unpromising candidates might hasten the identification and selection of promising candidates for chronic pain treatment, thereby helping to increase the likelihood of successful translation from the preclinical to clinical settings and reducing the high costs associated with their development. European Medicines Agency guidelines on the clinical development of medicinal products intended for the treatment of pain explicitly indicate that objective biomarkers might improve the development of drugs for chronic pain (EMA/CHMP/970057/2011). We postulate that this goal could be achieved by using a novel research paradigm taking advantage of improved objective measures of nociceptive signal processing, functional biomarkers of pain which translate between animals and humans, and pharmacokinetic-pharmacodynamic (PK-PD) modelling. In IMI2-PainCare-BioPain-RCT2, electrophysiological measures of spinal cord and brainstem reflex activity are used for these purposes.

The overall concept of the BioPain subtopic of IMI-PainCare (http://imi-paincare.eu) has been extensively described previously [2]. Briefly, we aim to assess the usefulness of selected candidate biomarkers at the peripheral, spinal, and central levels to assess drug exposure and target engagement to be used in the development of new analgesics. BioPain will analyze these functional pain biomarkers in healthy subjects and in preclinical species, where they will also be compared with standard behavioral assessments.

### Objectives {7}

BioPain has designed four placebo-controlled RCTs in healthy subjects with the objective of profiling four sets of pain biomarkers derived from non-invasive measures of peripheral nerve excitability (IMI2-PainCare-BioPain-RCT1)^1^, electrophysiological measures of spinal cord and brainstem reflex activity (IMI2-PainCare-BioPain-RCT2)^2^, electroencephalographic (EEG) measures of brain activity (IMI2-PainCare-BioPain-RCT3)^3^, and functional magnetic resonance imaging measures of brain activity (IMI2-PainCare-BioPain-RCT4)^4^, using three drugs registered as analgesics or known to act on the compartment of the nociceptive system, given as a single dose in four separate study periods: lacosamide acting preferentially on nociception at the peripheral level, pregabalin acting preferentially on nociception at the spinal level, and tapentadol acting preferentially on nociception at the supraspinal level.

IMI2-PainCare-BioPain-RCT2 will focus on biomarkers derived from non-invasive neurophysiological spinal and brainstem measurements. Specifically, it will evaluate (i) the RIII flexion reflex, which is a pure nociceptive spinal reflex widely used for investigating pain processing at the spinal level, sensitive to different experimental pain models and to the antinociceptive activity of drugs [3, 4]; (ii) the cervical N13 component of somatosensory evoked potentials (SEP), mediated by large myelinated non-nociceptive fibers and reflecting segmental postsynaptic response of wide dynamic range dorsal horn neurons at the level of cervical spinal cord [5, 6]; (iii) the R2 reflex, mediated by large myelinated non-nociceptive fibers, whose circuit consists of a polysynaptic chain lying in the reticular formation of the pons and medulla and whose recovery cycle is widely used to assess the brainstem excitability [7, 8]. Each neurophysiological measure will be used to relate to perceived pain intensity.

All four studies of the BioPain project will assess the effects of the medications on the biomarkers concurrently in a non-sensitized normal condition and a clinically relevant hyperalgesic condition. For this purpose, the trials will use high-frequency electrical stimulation (HFS) of the skin, a validated and non-invasive experimental procedure to induce, in healthy volunteers, a reversible but nevertheless sustained state of hyperalgesia due to sensitization of the nociceptive system [9, 10].

### Trial design {8}

IMI2-PainCare-BioPain-RCT2 is a multicenter, exploratory, single-dose, double-blind, randomized, placebo-controlled, 4-period, 4-way crossover, pharmacodynamic (PD), and pharmacokinetic (PK) study in healthy subjects.

**Fig. 1:**
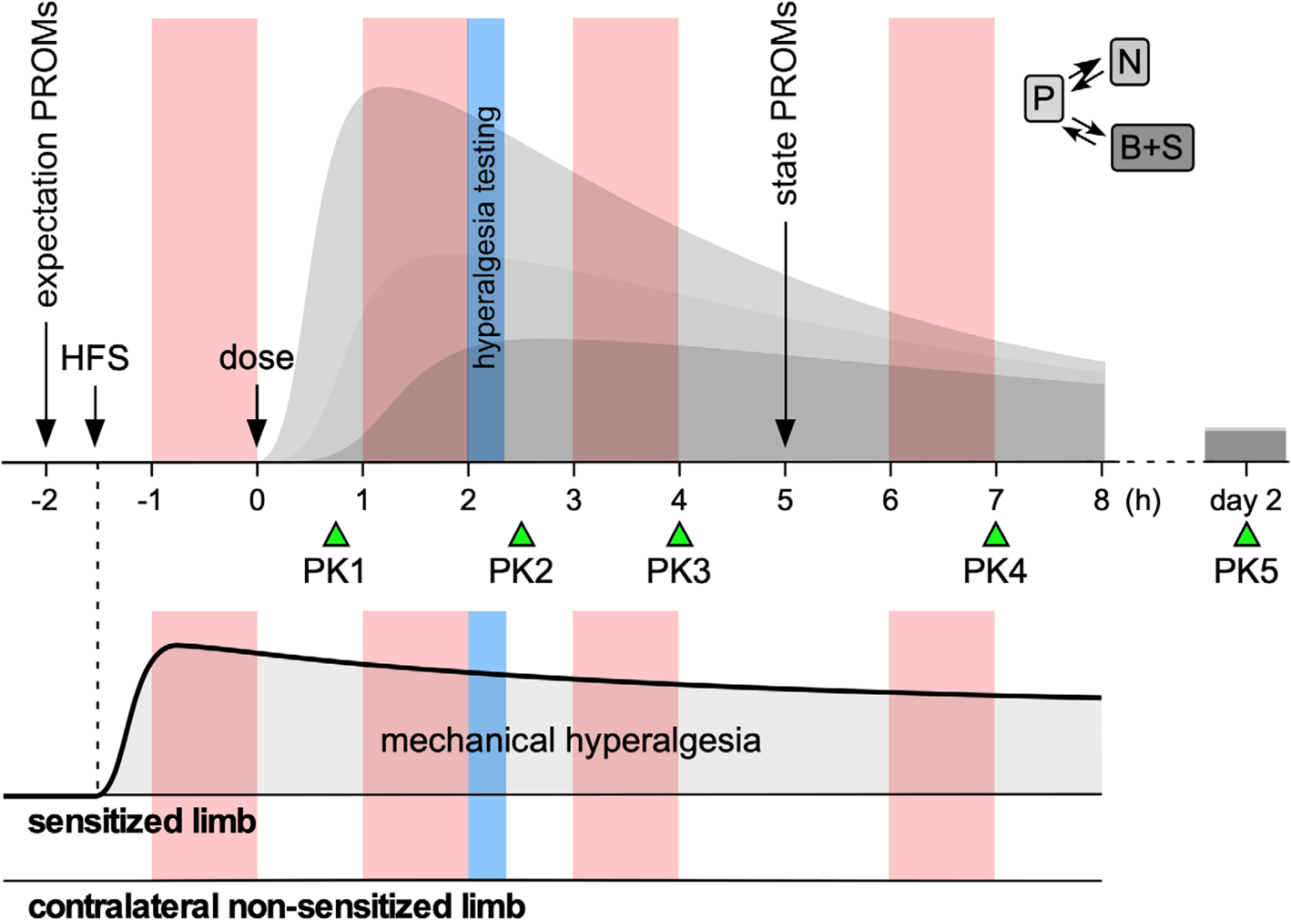
Trial design. The effects of a single oral dose of four different treatments (lacosamide, pregabalin, tapentadol, placebo) on spinal and brainstem biomarkers will be assessed in four separate study periods separated by at least 1 week. In each study period, five blood samples will be taken to model the pharmacokinetic (PK) profiles of the chosen drugs in plasma (P), peripheral nerves (N), spinal (S), and brain (B) compartments (theoretical PK curves shown in gray). After the induction of a hyperalgesic state using HFS, the biomarkers will be assessed at four time points shown in light red: before drug administration, and at three different times after drug administration, close to the expected maximum drug concentration and at relevantly lower drug concentrations. Patient-reported outcomes will be used to assess subject expectations (expectation PROMs) and state (state PROMs). Hyperalgesia testing (light blue) will be used to assess and compare the HFS-induced hyperalgesia across study periods and across the different BioPain RCTs. Reproduced from [2]

As shown in Fig. 1, the biomarkers will be evaluated by assessing the effects of three analgesic or antihyperalgesic agents (lacosamide, pregabalin, tapentadol) and placebo, given as a single dose in four separate study periods separated by at least 1 week.

In each study period, before drug administration and the first biomarker assessment, HFS will be applied consecutively to the left ulnar hand dorsum and to the dorso-lateral part of the left foot, in order to induce a sustained state of hyperalgesia restricted to the area of stimulation. This will allow evaluating drug effects on neurophysiological outcomes both for input conveyed within normal non-sensitized pathways (RIII flexion reflex elicited by stimulation of the non-sensitized limb and R2 recovery cycle) and for input conveyed within sensitized pathways (RIII flexion reflex and N13-SEP elicited by stimulation of the sensitized side).

Subsequently, the spinal and brainstem biomarkers will be measured four times: before drug administration, and at three time points expected to correspond to relevantly different drug concentrations.

The primary and key secondary endpoints of the study are the treatment-induced percentage change of the RIII flexion reflex area and the N13-SEP amplitude elicited by stimulation of the sensitized limb and the treatment-induced percentage change of R2 recovery cycle at different interstimulus time intervals after supraorbital nerve stimulation of the non-sensitized side.

Furthermore, as chronic pain is often accompanied by depression and anxiety, validated questionnaires will be used to assess psychological traits and states.

## Methods: subjects, interventions and outcomes

### Study setting {9}

Four clinical sites in four countries will participate: the Department of Neurology and Psychiatry of the Sapienza University of Rome in Italy (Principal Investigator: Andrea Truini), the Department of anesthesiology of the Cliniques universitaires Saint-Luc of the Université catholique de Louvain in Belgium (Principal Investigator: Patricia Lavand’homme), the Center for Biomedicine and Medical Technology Mannheim (CBTM) of the University of Heidelberg in Germany (Principal Investigator: Rolf-Detlef Treede), and the NeuroPain laboratory of the Center for Neuroscience Lyon of the Université Lyon in France (Principal Investigator: Luis Garcia Larrea). Details on the study sites can be obtained on the EudraCT clinical trials register (2019-000755-14).

All sites are academic hospitals and/or academic laboratories conducting research in human volunteers.

Furthermore, the following participants will assume non-clinical roles in this study:

- Heidelberg University Computing Center, Germany. Contribution: assuming responsibility for data storage and advanced statistical analysis.
- ConsulTech GmbH, Berlin, Germany. Contribution: ConsulTech will coordinate trial monitoring activities. Tasks include review and inspection of the quality of the data and the compliance to and implementation of regulations such as the Declaration of Helsinki, GCP and the Clinical trial plan.
- MRC Systems (spin-off from the University of Heidelberg and the German Cancer Research Center in Heidelberg), Germany. Contribution: MRC Systems will provide to each clinical partner the multipin electrode used to deliver HFS, as well as the mechanical pinprick stimulators for hyperalgesia testing.
- Pharmacometrics and Systems Pharmacology (PSP), Department of Pharmacy and Pharmaceutical Technology of the School of Pharmacy, University of Navarra, Spain. Contribution: Integrate from a quantitative mechanistic and translational perspective, PK/PD information gathered from the study, as well as PK/PD information provided by preclinical in vitro and in vivo studies conducted within the BioPain subtopic of IMI-PainCare. The end-product will consist of a model formulated on the basis on the known and data-driven mechanisms of action that can be (among several other applications) (i) used through modelling and simulation to optimize dosing scenarios and (ii) applied retrospectively or prospectively in other scenarios to get meaningful PK/PD parameters.
- Grünenthal GmbH, Aachen, Germany. Contribution: Co-leading the task to support consensus on final study designs across IMI2-PainCare-BioPain-RCT1 to RCT4. Co-leading the task of clinical study implementation and operations.
- Eli Lilly and Company, research site Arlington Square, UK. Contribution: Co-leading the tasks of data delivery and analysis (preclinical and clinical), and preclinical biomarker back-translation, including PK. PK/PD/pharmacometric co-leadership and analysis support.
- WELAB, Barcelona, Spain. Contribution: performing bio-analyses of the IMPs as laid down in separate specification manuals.
- Teva Pharmaceutical Industries Ltd., headquartered in Petah Tikva, Israel. Contribution: pharmacometric support, clinical programming, data collection and capturing, and input of expertise related to CDISC.

### Eligibility criteria {10}

Candidates’ eligibility will be assessed during an initial screening visit, where the compliance of the candidates with respect to the inclusion and exclusion criteria will be examined. After the screening visit, subjects will be either excluded from the trial or scheduled for the first study period. Tables 1 and 2 list the inclusion and exclusion criteria assessed during the screening visit. Since this is one in a set of four RCTs that are destined for a joined analysis, the inclusion criteria need to be harmonized across the four RCTs. Some inclusion/exclusion criteria, therefore, come from the needs of another one of the four studies (Table 1, item 3). The absorption of infrared laser radiation in RCT3 limits the recruitment to Caucasians; however, there are no evidence in the literature suggesting a different behavior of brainstem and spinal neurophysiological biomarkers depending on skin color.

**Table 1:** Inclusion criteria at screening visit (identical for the four RCTs)

	Inclusion criteria at screening visit	Justification / rationale
01	Provision of signed and dated informed consent form	Ethical requirement
02	Stated willingness to comply with all study procedures and regimens and availability for the duration of the study	Ethical requirement and to minimize dropout rate
03	Caucasian male or female subjects, aged 18 years to 45 years	To minimize variability. Laser heat stimuli used to elicit laser evoked potentials in RCT3 will be delivered to the skin using an Nd:YAP laser. Because skin reflectance, absorption and transmittance of the infrared radiations generated by this laser are highly dependent on skin pigmentation, only Caucasian participants with light skin will be recruited.^a^
04	Subjects must be in good health as determined by the medical history, physical and laboratory examinations and must not show any clinically significant deviations from reference ranges as determined by 12-lead electrocardiogram (ECG), vital signs (blood pressure, pulse rate and respiratory rate), and laboratory parameters (renal and hepatic function).	Subject safety and interpretability of results
05	Body mass index >18 kg/m^2^ and < 30 kg/m^2^ with a minimum body weight of 45.0 kg and a maximum of 100 kg (for men and women)	Consistent with being in good health
06	Ability to take oral medication	Practical reason
07	For female subjects of childbearing potential: use of highly effective contraception with a low failure rate defined as <1% per year for at least 1 month prior to screening and agreement to use such a method during study participation and for an additional 4 weeks after the end of study drug administration: - Combined (estrogen and progestogen containing) hormonal contraception, - An intra-uterine device (hormone-free), - Progestogen-only hormonal contraception associated with inhibition of ovulation, - An intra-uterine hormone releasing system (IUS) A woman of non-childbearing potential may be included if surgically sterile (i.e., after laparoscopic or hysteroscopic sterilization, hysterectomy or bilateral oophorectomy) or post-menopausal for at least 2 years.	To avoid pregnancies with potential harm to the unborn
08	Right hand dominance (assessed using the Edinburgh Handedness Inventory, and defined as a score ≥60)	To minimize variability

^a^This is one in a set of four RCTs that are destined for a joined analysis. The inclusion criteria needed to be harmonized across these RCTs. The requirement for Caucasians came from RCT3, due to absorption of infrared laser radiation

**Table 2:** Exclusion criteria at screening visit

	Exclusion criteria at screening visit	Justification / rationale
01	Presence of any medical devices (e.g., cardiac pacemaker), implants, or prosthesis unless it is beyond discussion that these will not put the subject’s safety during the study at risk and will not interfere with the results of the study.	To avoid interference with the purpose of the study and to ascertain the subject’s good health
02	Known or suspected allergic reactions or hypersensitivity to components of lacosamide (Vimpat®). Second or third degree atrioventricular (AV) block.	Contraindications for lacosamide
03	Known or suspected allergic reactions or hypersensitivity to components of pregabalin (Lyrica®).	Contraindications for pregabalin
04	Known or suspected allergic reactions or hypersensitivity to components of tapentadol (Palexia®). Known contraindication for drugs with μ-opioid agonist activity, i.e., significant respiratory depression, acute or severe bronchial asthma or hypercapnia. Present or suspected paralytic ileus. Acute intoxication with alcohol, hypnotics, centrally acting analgesics, or psychotropic drugs.	Contraindications for tapentadol
05	Not willing or able to abstain from changes in physical exercise activities during the study.	To avoid interference with the purpose of the study
06	Any chronic pain condition or recent (i.e., within the preceding 2 years) history thereof.	To avoid interference with the purpose of the study
07	Migraine (at least 1 attack in the last 24 months).	To avoid interference with the purpose of the study
08	Recurrent headache or back pain on more than 5 days/month in the last 3 months.	To avoid interference with the purpose of the study
09	Caffeine consumption of more than 8 servings of coffee, tea, or other caffeinated drinks per day. Each serving is approximately 120 mg of caffeine.	To avoid interference with the purpose of the study
10	Any relevant symptom of neurological dysfunction of the motor and sensory system that may interfere with the conduct of the study.	To avoid interference with the purpose of the study
11	Clinically evident psychiatric diseases (e.g., depression, anxiety).	To avoid interference with the purpose of the study
12	History or symptoms of central nervous system disease or peripheral nerve lesions or dysfunction with sequelae that may impact the study assessments or that may deteriorate by one dose of a drug with anti-epileptic, noradrenergic or opioid activity.	To avoid interference with the purpose of the study Subject safety
13	Focused neurological examination showing signs of abnormality.	To avoid interference with the purpose of the study
14	Active internal disease or sequelae of internal disease (e.g., diabetes mellitus, liver diseases, kidney diseases, cardiovascular diseases, hypo- or hyperthyroidism, hypertension).	To ascertain the subject’s good health
15	Diseases or conditions known to interfere with the distribution, metabolism, or excretion of drugs.	To avoid artifacts
16	Clinically significant disease (e.g., medical history of infection with human immunodeficiency virus (HIV) Type 1 or Type 2, hepatitis B, or hepatitis C or condition that may affect efficacy or safety assessments, or any other reasons which, in investigator’s opinion, may preclude the subject’s participation in the trial.	Safety of investigator and their staff Standardization of the trial population
17	Not willing or able to abstain from alcohol from 48 h prior to any study period and until the end of the study period.	To ascertain and protect the subject’s good health and suitability for the study
18	Consumption of cannabis in the last 4 weeks prior to the study.	To ascertain and protect the subject’s good health and suitability for the study
19	Evidence or history of alcohol or drug (opioids, amphetamines, benzodiazepines cannabinoids) abuse (as defined by ICD-10 or DSM IV) including positive or missing drugs of abuse screen (urine drugs of abuse test). Consumption of more than 21 alcohol units per week for male subjects and more than 14 units per week for female subjects (1 alcohol unit = 1 beer [12 oz/355 mL] = 1 wine [5 oz/150 mL] = 1 liquor [1.5 oz/40 mL] = 0.75 oz/20 mL alcohol).	To ascertain and protect the subject’s good health and suitability for the study
20	Habitually smoking more than 10 cigarettes, 2 cigars, or 2 pipes of tobacco per day within the last 6 months before enrollment in this trial.	To ascertain the subject’s good health
21	Known or suspected of not being willing or able to comply with the requirements of the trial protocol or the instructions.	The investigator will specifically investigate the presence of any uncertainties of the subject and whether he/she correctly understood the study requirements, to ascertain the suitability for the study.
22	Inability to communicate meaningfully with the trial site staff (e.g., insufficient language skills), highlighted during the interview with the investigator.	To ascertain the subject’s safety
23	Any person with direct involvement in the trial conduct; any person under the direct supervision of the investigator or dependent on the investigator.	Ethical requirement
24	Blood loss of 500 mL or more (e.g., owing to blood donation) within 3 months before enrollment in this trial.	To ascertain the subject’s suitability for the study
25	Pregnancy, planned pregnancy, or lactation.	Ethical requirement to protect the unborn or newborn child, given the potential teratogenicity of the drugs used.
26	Presence of dermatological conditions in the test areas of the study that would prevent the proper application of study procedures, such as electrodes for HFS, pinprick (dermatitis, psoriasis, contact eczema, local changes of the skin due to regularly playing volleyball, etc.).	To avoid interference with the purpose of the study
27	Any other reason to exclude the subject according to judgment by the investigator	To avoid interference with the purpose of the study, the investigator is free to rely on his/her clinical experience to assess the suitability of the subject.
A set of *temporary exclusion criteria* have also been defined. The subject will not be excluded if some of these temporary exclusion criteria are met during the screening visit. Instead, the first study period may be postponed. Before the start of the first study period, previously met temporary exclusion criteria will be checked again, and their absence will be verified before the screening for the first study period takes place.		
28	Any drug intake in the past 2 weeks including antibiotics, herbal medicines and other remedies except the following allowed drugs: oral paracetamol or ibuprofen for a self-limiting condition (e.g., toothache, bruise) for up to 3 days in total within the past 2 weeks; oral antihistaminics and nasal aerosol and topical treatments for seasonal allergy up to 1 week before screening; contraceptives are allowed without time limit.	To ascertain the subject’s good health and to avoid interference with the purpose of the study
29	Any transient illness within 2 weeks before screening.	To ensure the subject’s good health
30	Changes in physical exercise activities, e.g., starting workout/training within 1 week before screening.	To avoid interference with the purpose of the study
31	Current or recent (during the preceding 2 weeks) acute pain lasting more than 4 h.	To avoid interference with the purpose of the study
32	Jet lag / irregular working hours / sleep restriction in the last 3 days before the screening period.	To avoid interference with the purpose of the study

### Who will take informed consent? {26a}

Before carrying out any trial-related procedure, freely given informed consent will be obtained by authorized trial site staff. The informed consent is identical to all four RCTs, and it has been previously described [2]. Informed consent will be obtained by the Principal Investigator or an appropriately trained delegate before any trial-related procedures following the GCP guidelines and applicable regulatory requirements. Each study subject will be fully informed of all pertinent aspects of the trial. The subjects will sign and date the informed consent form themselves after having sufficient time to make their decision on participating in the trial. Each participant will receive a copy of the signed consent form. The information sheet and informed consent form have been approved by the relevant Independent Ethical Committees (IECs). Data privacy details will be part of the subject’s information and informed consent forms which will be subject to ethical and legal review by the appropriate Ethics Committees prior to commencement of the study. The rules on data privacy will apply not only to data themselves but also to cover biological samples of the subjects that are collected and processed in the course of the proposed trial.

### Additional consent provisions for collection and use of participant data and biological specimens {26b}

The informed consent will explain the possibility that anonymized samples and data could be shared by the Investigator with third parties, naming these parties, specifying the samples and data to be shared, and explicitly obtaining the subjects’ consent for this sharing.

## Interventions

### Explanation for the choice of comparators {6b}

The objective of IMI2-PainCare-BioPain-RCT2 is to test whether parameters derived from spinal and brainstem neurophysiological measurements obtained in healthy volunteers exposed to a single dose of a drug can be used to evaluate the dynamic effects of a given drug on nociceptive signal processing and thereby be used as pharmacodynamic biomarkers for analgesic drug development.

In line with the common design of pharmacodynamic studies in healthy subjects, the single-dose, crossover design is adequate for the studies of the BioPain project. Randomization, blinding, and placebo control serve to minimize bias.

The Rationale for the chosen investigational medicinal products (IMPs) and their dose have been described previously for RCT3 [2] and will be briefly summarized here.

#### Rationale for the chosen investigational medicinal products (IMPs) and their dose

##### Lacosamide

In the EU, lacosamide has been approved as monotherapy or add-on therapy for epilepsy. In 2008, UCB Pharma withdrew its application for a marketing authorization for lacosamide for the treatment of painful diabetic polyneuropathy. Lacosamide is efficacious in animal models of neuropathic pain [11]. Studies in patients obtained inconsistent results [12–19]. In 2 out of 3 studies, statistically significant superiority over placebo was found for the 200 mg BID regimen, although at the cost of a 12.2% rate of premature discontinuations. The rate of discontinuations almost doubled from the 200 mg BID to the 300 mg BID regimen and is the most likely cause for the failure of this dose to achieve superiority over placebo. Despite the high rate of discontinuations related to adverse events, no specific safety issue of lacosamide became apparent from these studies as confirmed by the European Withdrawal Assessment Report (EMEA/CHMP/658067/2008) that accompanied the rejection by the EMA of a marketing authorization of lacosamide for neuropathic pain. Recently, a double-blind RCT has shown the effect of lacosamide in patients with nav1.7 mutations-related small fiber neuropathy [16] and a study in painful small fiber neuropathy found that lacosamide normalized the firing pattern of C fibers using microneurography, reduced heat and pain thresholds, and also reverted abnormal excitability of nociceptors derived from human-induced pluripotent stem cells, suggesting a specific modification of the function of peripheral nociceptors [17]. In conclusion, there is adequate evidence that a 200-mg dose of lacosamide has a relevant analgesic effect, with acceptable side effects.

##### Pregabalin

In the EU, pregabalin has marketing authorization for the treatment of peripheral and central neuropathic pain in adults. The dose range is 150 to 600 mg per day given in either two or three divided doses. Single oral doses of 300 mg pregabalin have been administered to almost 200 healthy subjects [20–23]. The intensity of AEs ranged from mild to severe. Most frequently occurring AEs were dizziness, somnolence, fatigue, and euphoric mood. No subject was withdrawn from the study for safety reasons. There is adequate evidence that a 150-mg dose of pregabalin has a clinically relevant analgesic effect. In conclusion, a single dose of 150 mg pregabalin seems an acceptable single dose for a biomarker study in healthy subjects.

##### Tapentadol

Tapentadol sustained release is indicated in the EU for the management of severe chronic pain in adults, which can be adequately managed only with opioid analgesics. Tapentadol immediate release (film-coated tablets) is indicated in the EU for the relief of moderate to severe acute pain in adults, which can be adequately managed only with opioid analgesics. Two randomized withdrawal trials indicated effect of tapentadol for painful diabetic polyneuropathy [24, 25]. One-hundred-milligram tapentadol administered to healthy subjects are referred to as highest therapeutic doses.

Lacosamide, tapentadol, and pregabalin have a potential teratogenicity; for this reason, pregnancy is a mandatory exclusion criterion.

##### Placebo

Placebo serves to minimize bias and to act as a control.

#### Rationale for the induction of hyperalgesia using high-frequency stimulation (HFS)

The Rationale for the induction of hyperalgesia using high-frequency stimulation (HFS) have been described previously for RCT3 (2) and will be briefly summarized here.

High-frequency electrical pulses are delivered to the skin using a multipin electrode designed to preferentially activate cutaneous nociceptors (HFS Electrode “EPS-P10”, MRC Systems GmbH, Heidelberg, Germany) composed of a rectangular anode (area: 24 × 20 mm^2^) and a cathode (diameter: *Ø* = 21 mm) equipped with 10 needle pins arranged on a circle with a diameter of 5 mm. It is a validated and non-invasive procedure to induce a hyperalgesic condition [9]. Numerous studies showed that cutaneous HFS delivered using this electrode (e.g., 100-Hz trains lasting 1 s of 2-ms electrical pulses, repeated 5 times at an intensity sufficient to generate strong activity in small-diameter nociceptive afferents) leads to a stable hyperalgesia [9, 10] lasting at least 4 h [26] and a marked secondary hyperalgesia to mechanical pinprick stimulation due to central sensitization. HFS does not generate any confounding long-lasting spontaneous after sensation and induce a local skin flare response, indicative of neurogenic inflammation that can be exploited to monitor peripheral sensitization effects [9].

### Intervention description {11a}

The IMP description is the same as described in the protocol for our related trial [IMI2-PainCare-BioPain-RCT3] see [2] for more details.

For the induction of hyperalgesia, HFS will be delivered to superficial nerve terminals using a multipin surface electrode similar to the electrode used in Klein et al. [9] and developed by MRC. The stimuli will be applied to the skin of the left ulnar hand dorsum and the dorsolateral side of the left foot. The electrical pulses will be generated by a standard, CE-approved, constant-current electrical stimulator routinely used for clinical diagnostic purposes. The stimulation will consist in trains of 100-Hz pulses lasting 1 s and repeated five times, at an intensity sufficient to generate strong activity in small-diameter nociceptive afferents.

### Criteria for discontinuing or modifying allocated interventions {11b}

At the beginning of each of the four study periods, subjects will undergo a new compliance assessment: if any of the criteria listed in Table 3 should occur, the subject will be excluded from the study period.

**Table 3:** Exclusion criteria at study periods reproduced from [2]

	Exclusion criteria at study periods	Justification / rationale
33	For female subjects of child bearing potential: positive or missing pregnancy test	To protect a fetus
34	Positive or missing urine test for drugs of abuse (opioids, amphetamines, benzodiazepines, cannabinoids).	Subject safety and to avoid interactions with, e.g., tapentadol (PD interactions, safety interactions)
35	Blood loss of 500 mL or more (e.g., owing to blood donation) since screening.	To ascertain the subject’s suitability for the study
36	Any other reason to exclude the subject according to judgment by the investigator	To avoid interference with the purpose of the study.
Temporary exclusion criteria at study periods. The subject is not excluded if some of these temporary exclusion criteria are met at screening of the study period. Instead, the study period may be postponed. If this is the case, all temporary exclusion criteria will be checked again.		
37	Alcohol consumption in the last 48 h prior to the study period.	Subject safety and to avoid interactions with, e.g., tapentadol (PD interactions, safety interactions)
38	Intake of any drug including herbal medicines and other remedies except the following: contraceptives; oral paracetamol or ibuprofen up to the maximum recommended dose according SmPC, with last intake for both >4 days prior to each study period for a self-resolving condition.	As described for screening visit
39	Changes in physical exercise activities, e.g., starting workout/training within 1 week prior to the study.	To avoid interference with the purpose of the study
40	Current pain within the last 4 days before the study period.	To avoid interference with the purpose of the study
41	Any transient, clinically relevant illness within 4 days before the period.	To ensure the subject’s good health
42	Incidentally not willing or able to comply with the requirements of the trial protocol or the instructions or to communicate meaningfully with the trial site staff.	To ascertain the subject’s suitability for the study
43	Incidentally unable to take oral medication.	Requirement for the study
44	Jet lag / irregular working hours / sleep restriction in the last 3 days before the period.	To avoid interference with the purpose of the study

### Strategies to improve adherence to interventions {11c}

At each study period, a single oral dose of lacosamide, pregabalin, tapentadol, or placebo will be taken with 100 mL of plain water. After intake of the study medication, the investigator will inspect the subject’s mouth to verify that the medication has been swallowed.

### Relevant concomitant care permitted or prohibited during the trial {11d}

Prohibited drugs at screening and for each study period are described in Table 2 (Exclusion Criterion #28) and 3 (Exclusion Criterion #38), respectively. If these temporary exclusion criteria are met, the study period will be postponed.

### Provisions for post-trial care {30}

The provisions for post-trial care are the same as described in the protocol for our related trial [IMI2-PainCare-BioPain-RCT3] see [2] for more details. Briefly, IMI2-PainCare-BioPain-RCT2 is a study conducted in healthy volunteers. No post-trial care is thus foreseen. A follow-up telephone call will be made between 7 and 14 days after the last study period to ensure the absence of adverse events. At each participating site, the Principal Investigator will arrange suitable insurance for the subjects included in this trial.

If changes to the trial are implemented after the initial insurance was arranged, e.g., due to protocol amendments, the Principal Investigator will notify the insurance company of these changes in accordance with the insurance conditions.

### Outcomes {12}

The objective of the study is to evaluate the effect of the study drugs on a set of biomarkers derived from spinal and brainstem neurophysiological measurements.

#### Rationale for the chosen biomarkers

All biomarkers tested in BioPainRCT2 are derived from non-invasive neurophysiological measurements of spinal and brainstem excitability (RIII reflex, N13-SEP, R2 recovery cycle). RIII flexion reflex is a polysynaptic response corresponding to the withdrawal of the stimulated limb and resembles the hind-paw flexion reflex in animals. The flexion reflex in both animals and humans is a pure nociceptive reflex mediated by a complex circuitry modulated at spinal and supraspinal level. The correlation between the pain threshold and RIII reflex threshold, the pain intensity stimulus-response curve, and the reflex size stimulus-response curve suggest that the RIII component of the flexion reflex might be used as an “objective” measure of experimental pain in humans. Several studies showed that this reflex response is sensitive to different experimental pain models and to the antinociceptive activity of drugs [3, 4].

N13-SEP is a spinal evoked potential, elicited by electrical stimulation of the ulnar nerve at the wrist. It is mediated by large myelinated non-nociceptive fibers and reflects segmental postsynaptic response of dorsal horn interneurons at the level of cervical spinal cord [5, 6]. Given that HFS modulates excitability of the dorsal horn, we hypothesize that the experimental pain models may affect N13-SEP amplitude.

R2 blink reflex, elicited by electrical stimulation of the supraorbital nerve, is mediated by large myelinated non-nociceptive fibers. The reflex circuit consists of a polysynaptic chain lying in the reticular formation of the pons and medulla [7, 8]. The recovery cycle of R2 is widely used for assessing the brainstem excitability.

#### Primary and key secondary outcomes

The two primary and the three key secondary endpoints (Table 4) are the treatment-induced percentage change of the RIII flexion reflex area and the N13-SEP amplitude elicited by stimulation of the sensitized limb and the treatment-induced percentage change of R2 recovery cycle at different interstimulus time intervals after supraorbital nerve stimulation of the non-sensitized side.

**Table 4:** Primary and key secondary endpoints

**Primary endpoints:**	
1. To test if the percentage of change of the RIII area of the flexion reflex at the time point t60 post-drug administration vs the pre-drug time point, differs in the tapentadol period as compared to the placebo period, at the sensitized lower limb.	
2. To test if the percentage of change of the N13-SEP amplitude at the time point t60 post-drug administration vs the pre-drug time point, differs in the tapentadol period as compared to the placebo period, at the sensitized upper arm.	
**Key secondary analyses of the primary endpoints:**	
1. To test if the percentage of change of the RIII area of the flexion reflex at the time point t60 post-drug administration vs the pre-drug time point, differs in the pregabalin and/or lacosamide period as compared to the placebo period, at the sensitized lower limb.	
2. To test if the percentage of change of the N13-SEP amplitude at the time point t60 post-drug administration vs the pre-drug time point, differs in the pregabalin and/or lacosamide period as compared to the placebo period, at the sensitized upper arm.	
3. To test if the percentage of change of the R2 recovery cycle at 500 ms interstimulus time interval at the time point T60 post-drug administration vs the pre-drug administration differs in the tapentadol, pregabalin and/or lacosamide periods as compared to the placebo period.	

#### Definition of other pre-specified analyses

The following secondary endpoints will be investigated and compared across the four treatment groups:

- The percentage of change in the RIII threshold across all post-drug time points vs. the pre-drug baseline time point, at the sensitized limb
- The percentage of change in the intensity of the sensation elicited by the electrical stimulation for the RIII reflex recording, as assessed with the NRS 0-100 points, at time point T+60 min post-drug administration vs. the pre-drug time point, at the sensitized limb.
- The percentage of change of the RIII area of the flexion reflex at the time point t60 post-drug administration vs the pre-drug time point, at the non-sensitized limb.

Additional exploratory endpoints will be:

- The percentage of change of the R2 recovery cycle at 250 and 750 ms interstimulus time interval.
- The N13-SEP and R2 latency changes.
- RIII flexion reflex changes at the sensitized and non-sensitized side (area, threshold, perception) across the four PD time points in the four study periods.
- Somatosensory evoked potential variables changes (amplitude and latency of N9, N13, and N20) across the four PD time points in the four study periods.
- Changes in amplitude ratios of somatosensory evoked potentials (N13/N9, N13/N20, N20/N9) across the four PD time points in the four study periods
- R2 recovery cycle at the different interstimulus time intervals, across the four PD time points in the four study periods
- Gender differences of neurophysiological and hyperalgesia variables
- Correlation between subject’s level of anxiety and expectation of pain and PD1 neurophysiological variables
- Correlation between subject’s expectation of pain relief and hyperalgesia variables
- PK/PD analysis. As both drug concentrations and biomarker responses are measured at several time points post-drug administration, the relationship between drug levels and select biomarkers will be explored and modelled.

### Participant timeline {13}

**Fig. 2:**
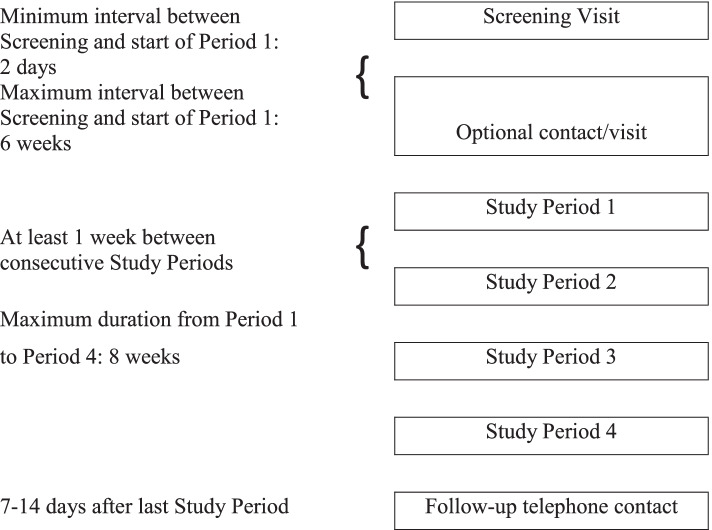
Timeline of the study which includes a screening visit, an optional contact, four study periods separated by at least 1 week, and a follow-up telephone contact

The participant timeline is the same as described in the protocol for our related trial [IMI2-PainCare-BioPain-RCT3] see [2] for more details. Briefly, Fig. 2 summarizes the participant timeline which includes a screening visit, followed by four study periods and a follow-up telephone contact similar to RCT3 and 1 [2]. There will be an optional contact before the first study period. If screening of exclusion criteria for eligibility for Period 1 shows that one or more temporary exclusion criteria are met, the start of Period 1 can be postponed and re-scheduled [2]. Each subject is expected to be in the trial for approximately a minimum of 30 days and a maximum of 14 weeks.

#### Screening visit

As described previously for the RCT3 [2], the following procedures will be performed:

- Explain the purpose of the research, the extent and burden of the procedures and assessments.
- Obtain informed consent.
- Assess subject handedness using the Edinburgh Handedness Inventory.
- Record demographic data.
- Record prior and concomitant medication.
- Record clinically relevant medical and surgical history.
- Assess inclusion criteria.
- Perform a focused neurological examination in the presence of any clinically evident sensory disorder and a physical examination if indicated from the medical history.
- Record a 12-lead electrocardiogram and verify absence of signs of second or third degree atrioventricular block.
- Perform urine pregnancy test.
- Collect a blood sample to verify normal renal and hepatic functions.
- Perform urine test for drug abuse (opioids, amphetamines, cannabinoids) and perform alcohol consumption check.
- Record psychosocial characteristics using patient-reported outcome measures and validated questionnaires.
- Instruct the subject on the study-specific procedures including how to use the rating scales.
- Demonstrate the test stimuli that will be used, induce sensitization at the left hand dorsum using HFS, and perform hyperalgesia testing 20 min after induction.
- Assess exclusion criteria specific for the screening visit.

#### Optional contact before start of treatment period

The optional (telephone / email) contact may occur after the screening visit and at latest 48 h before the first study period, to arrange for the subject to attend the first study period, and remind the subject to abstain from alcohol during 48 h and from drug intake in the 4 days preceding each study period.

#### Treatment periods: study periods 1, 2, 3, and 4

Each study period will be separated by at least 1 week. Subjects will have a light breakfast at home. The schedule of events is identical for all study periods and is provided in Table 5 and presented previously for RCT3 [2].

**Table 5:**
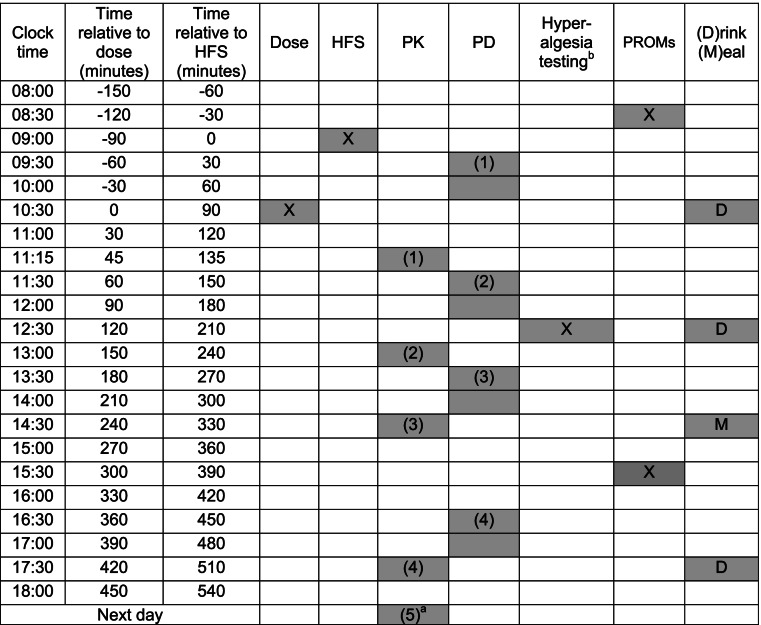
Detailed timetable of procedures and assessments in periods 1, 2, 3, and 4

^a^The PK sample on next day can be taken at any suitable time provided that the exact time of sampling is precisely recorded

^b^Hyperalgesia testing at the sensitized and contralateral size, harmonized across all four IMI2-PainCare-BioPain RCTs

Briefly, procedures, assessments, and events during a period are as follows:

- The subject will have breakfast at home and arrive at the site at or before 08:00 AM.
- Record prior and concomitant medication.
- Urine screening test for drugs of abuse and alcohol consumption check.
- For female subjects: urine pregnancy test.
- Reassess subject eligibility for the study according to inclusion and exclusion criteria.
- Train / instruct again the subject on the study-specific procedures.
- Complete PROMs assessing subject expectations.
- Optionally, according to local practices, an indwelling venous catheter will be inserted at the start of each study period and will be left in place for the duration of the study day.
- HFS will be applied to the ulnar left hand dorsum and to the dorsolateral part of the left foot to induce sensitization.
- IMP administration.
- A total of 5 blood samples (6 mL each) will be taken as scheduled in Table 5 for pharmacokinetic (PK) analyses. The last sample will be taken on the next day, at any suitable time.
- One pre-dose and 3 post-dose pharmacodynamic (PD) biomarker assessments will be made as scheduled in Table 5.
- Hyperalgesia testing at the sensitized and contralateral side will be made as scheduled in Table 5, harmonized across all four IMI2-PainCare-BioPain-RCTs.
- Complete PROMs assessing tiredness and anxiety.
- Drinks (water or sugared juice, e.g., apple juice) and a light meal will be served as scheduled in Table 5.
- Instruct the subject not to drive or bike or operate machinery for the entire day (risk of sedation or dizziness caused by IMP). Instruct the subjects that they should not drive or bike or operate machinery on the following day if they feel drowsy or dizzy.
- Upon leaving the trial site, if the subject is feeling drowsy or dizzy, arrange for the participant to be driven home by taxi.

#### Follow-up telephone call

Between 7 and 14 days after the end of the last study period, the absence of untoward medical or mental sequelae of the study will be ascertained in a follow-up telephone call with the subject.

### Sample size {14}

Given the rationale of improving preclinical to clinical translation of pain effects and the intention to use these biomarkers in future early clinical pharmacology studies, a robust signal in a small number of subjects is required.

Previous studies have investigated the effect of different drugs on the area of the RIII reflex. In particular, some studies have assessed the effect of opioids on the RIII area [27, 28]. A controlled study based on 12 subjects showed that the RIII flexion reflex threshold and area after IV administration of 0.002 mg/kg buprenorphine IV changed by 14.1 units (SD = 17.5) corresponding to 53.7% (SEM 20.2%) in relative terms [28]. With these characteristics, a study evaluating 32 subjects with a paired *t*-test would have a power of approx. 90% to detect group differences as significant. In another study, doses of 0.1, 0.2, and 0.3 mg/kg morphine IV produced a 20%, 45%, and 70% depression of the RIII flexion reflex in 6 healthy subjects [27]. Using the 0.1 mg/kg arm as an example, SD was roughly estimated to be 37 from the study’s graphical summary of results. Based on previous findings, a study evaluating 40 or 44 subjects with a paired *t*-test would have a power of approx. 70 to 75% to detect group differences as significant. We expect that 100 mg of tapentadol may show similar effect characteristics on the RIII reflex area.

Unfortunately, no reliable quantitative information is currently available on the effect of drugs on N13-SEP and R2 recovery cycle.

To compensate for drop-outs during the first period, it is recommended to enroll and randomize a total of 56 subjects into the treatment phase. It is recommended to aim for sample sizes that are balanced across centers (e.g., 14 per center if 4 centers are involved).

It should also be noted that the insights into the characteristics of the investigated drugs and biomarkers (cf. PK/PD analyses and/or test-retest reliability) also contribute to the trial rationale irrespective of any significant finding in the primary analysis.

### Recruitment {15}

The recruitment is the same as described in the protocol for our related trial [IMI2-PainCare-BioPain-RCT3] see [2] for more details.

## Assignment of interventions: allocation

### Sequence generation {16a}

Randomization is done blind to investigators as described previously [2] and will be by site.

Subjects will be enrolled by the participating sites and assigned to intervention sequence according to enrolment number per study site.

The randomization list (per site) will be built in blocks of four “4-period sequences,” these sequences being random permutations of the four 4-period sequences of a (basic) Latin square. Each block (allowing the sequential allocation of four subjects) will use an own (new) random permutation of the Latin squares’ four rows.

At the first study period day, before first IMP administration, subjects will be randomized to receive the lowest available randomization number at the site.

### Concealment mechanism {16b}

Each IMP will be administered overencapsulated as single oral dose (two capsules).

A sealed decoding envelope per treatment period will be provided for each randomization number. Each envelope will contain the identification of the IMP allocated to that subject.

### Implementation {16c}

The implementation is the same as described in the protocal for our related rial [IMI2-PainCare-BioPain-RCT3] see [2] for more details. Briefly, the IMPs will be purchased centrally by the Heidelberg University Hospital Pharmacy, which will perform the manufacturing of placebo capsules, the overencapsulation, the packaging, and the labeling of the IMPs, in compliance with applicable local regulations. Detailed information about the packaging and labeling is laid down in a specification document (available on request). The Heidelberg University Hospital Pharmacy will also provide the allocation sequence and sealed envelopes.

Storage conditions will be specified on the labels of the IMPs according to applicable EU and local regulation. The IMPs will be stored in a secure place with restricted access and temperature monitoring. The IMP delivery (shipping), return, and disposal will be performed in accordance with the specification document (available on request). All sites will be licensed according to local law for the receipt, storage, handling and administration of narcotics.

General unblinding (by the statisticians) will only take place after the trial has been completed and the database is locked, except for single-subject unblinding in emergency situations.

## Assignment of interventions: blinding

### Who will be blinded {17a}

As described for RCT3 [2], the investigator/trial personnel and subjects will be blinded to the assignment of pregabalin, tapentadol, lacosamide, and placebo (double-blind procedure).

The personnel analyzing the plasma samples for PK analysis will be unblinded during the bioanalytical analysis but will supply their data to the trial database in a blinded fashion.

### Procedure for unblinding if needed {17b}

Each envelope contains the identification of the IMP allocated to that subject. The investigator may only break the code (open the envelope) when it is necessary and in the subject’s interest to identify the given IMP. If a sealed decoding envelope needs to be opened in an emergency case, the reason and time for unblinding as well as the staff member performing or informed of the unblinding is documented.

If required by local regulations, the IEC needs to be informed.

## Data collection and management

### Plans for assessment and collection of outcomes {18a}

#### Collection of pharmacokinetic data

The collection of pharmacokinetic data is the same as described in the protocol for our related trial [IMI2-PainCare-BioPain-RCT3] see [2] for more details.

#### Collection of demographic data and other baseline characteristics

As detailed for RCT3 [2], investigators will collect demographic data, concomitant medication, and medical history and will check for abstinence of alcohol consumption.

At the screening visit, investigators will perform a focused neurological examination in the presence of any clinically evident sensory disorder, a 12-lead electrocardiogram (ECG) to specifically check for signs of 2nd or 3rd degree atrioventricular (AV) block, a urine test to check for pregnancy and drug abuse and a blood sample will be collected to verify normal renal and hepatic functions.

#### Collection of pharmacodynamic data

All participating centers have the equipment and expertise required for acquisition of the pharmacodynamic data. Each sponsor will provide a detailed operational manual for the collection of PD data.

#### RIII recording

Electrical stimulation of the sural nerve evokes a flexion reflex in the lower limb consisting of an early, inconstantly present component, called the RII reflex and a late, stable, response, the RIII reflex. RIII reflex is a pure nociceptive reflex, mediated by A-delta fibers, and corresponding to the (subclinical) withdrawal of the stimulated limb.

The RIII reflex will be evoked after sural nerve stimulation at the lateral malleolus using a train of five square electrical pulses delivered at a stimulus frequency of 200 Hz, randomly applied every 15–20 s (to avoid habituation). The stimulus intensity used to evoke the reflex will correspond to 1.5× the threshold of the RIII component. The RIII threshold will be defined as the stimulation intensity generating stable reflex responses at a rate of ≥60% after a series of 20 stimuli [3].

The RIII reflex will be recorded in the sitting position, and subjects will be relaxed with their lower limbs positioned to achieve complete muscle relaxation, with the knee flexed at 130° and the ankle around 90°. The RIII reflex will be recorded with standard surface electrodes placed over the short head of the biceps femoris, after carefully cleaning the skin. The recording electrode will be positioned proximally, on the belly of the short head of the biceps femoris muscle, and the reference electrode will be placed on the biceps tendon. Twelve responses will be recorded. Verbal NRS ratings of pain intensity will be collected after each stimulus and after the entire block (0 = no pain at all, 100= most intense imaginable pain).

The RIII onset latency varies between 85 and 130 ms. Responses with latencies between 40 and 60 ms should be classified as the early non-nociceptive RII response. The RIII amplitude will be measured as the area under the biceps femoris muscle EMG curve, between 80 and 130 ms. The area under the EMG curve must be measured for each trial.

#### N13 recording

The N13 spinal evoked potential will be elicited by electrical stimulation of the ulnar nerve at the wrist. The cathode will be placed 2 cm proximal to the wrist crease, the anode on the wrist crease. The ulnar nerve will be stimulated using electrical pulses of 0.1 ms of duration, with a frequency of 4 Hz. The intensity will be set at the threshold for evoking muscle twitch in the ulnar nerve muscles of the hand. The N13 will be recorded with posterior spinal cervical electrode placed over the 6th cervical spinous process (Cv6). The reference will be on the anterior neck at the level of the glottis on the midline (AC). The ground electrode will be placed on the stimulated limb between the stimulation site and the recording electrode. Muscle artifacts will be eliminated by making the subject as comfortable as possible. The subject will lie on a medical cot in a complete relaxed position. Two blocks of 650 trials will be collected, superimposed, in order to evaluate the reproducibility, and averaged. The N13 amplitude will be measured from the positive peak of the P9 to the negative peak of the N13 (when the P9 is not clearly identified, the amplitude will be measured from the isoelectric line).

#### Additional SEP components

N9 is a near-field potential generated by afferent action potentials in the brachial plexus. Electrodes will be placed bilaterally, over the Erb’s point, within the angle formed by the posterior border of the clavicular head of the sternocleidomastoid muscle and the clavicle, 2–3 cm above the clavicle. The recording electrode ipsilateral (EPi) to the site of stimulation and the reference electrode contralaterally (EPc). The N9 is the principal negative peak seen in the Epi-EPc channel. The amplitude of N9 will be measured on the Epi-EPc channel from its peak to that of the initial positive deflections.

N20-P25 is generated in the primary somatosensory cortex, in the posterior bank of the central sulcus. The locations of scalp electrodes will be specified using the 10–20 International system of EEG electrode placement. Parietal scalp electrodes will be positioned 5 cm posterior to Cz and 7 cm lateral to midline, contralateral to the site of stimulation (Pc). Reference electrode will be placed on Fz. N20 represents the largest early negative deflection at Pc. The N20 peak is usually identified as a portion of the negative potential just preceding the sharp drop-off toward the succeeding cortical positive peak P25. The P25 component will be recognized as the main prominent positive peak succeeding the N18-N20 complex at Pc. The amplitude of the cortical potentials will be measured from the N20 peak to the P25 on the Pc-Fz channel.

#### R2-blink reflex recording

The blink reflex is a brainstem reflex, mediated by large myelinated non-nociceptive fibers, consisting of an early component, the R1, and a late component, the R2, mediated by a polysynaptic circuit lying in the brainstem reticular formation.

The blink reflex will be elicited by electrical stimulation of the supraorbital nerve. To record blink reflex, the subject will lie on a medical cot in a relaxed position, with the gaze down. The supraorbital nerve will be stimulated immediately above the supraorbital notch. The EMG signals will be collected from the orbicularis oculi muscle bilaterally. The recording electrode will be placed over the muscle, the reference electrode at the lateral angle of the eye. Five responses will be recorded. The R2 amplitude will be measured as the area under the muscle EMG curve between 25 and 85 ms. The recovery cycle of the R2 will be investigated with paired stimulation of the supraorbital nerve (using the same stimuli parameters applied for the blink reflex recording). Interstimulus time intervals of 250, 500, and 750 ms will be used. For each interstimulus time interval, seven trials will be collected.

The recovery cycle will be then calculated by expressing the area of the response to the second shock (test) as a percentage of the area of the response to the first shock (conditioning).

#### Non-neurophysiologically derived pharmacodynamic data

The non-neurophysiologically-derived pharmacodynamic data are the same as described in the protocol for our related trial [IMI2-PainCare-BioPain-RCT3] see [2] for more details. Briefly the following data will be collected:

##### Pain intensity and unpleasantness ratings

The subjects will be also asked to rate the average unpleasantness/pain of each PD block using a 101-point unpleasantness/pain rating scale where 0 is defined as “not unpleasant/painful at all” and 100 is defined as “extremely unpleasant/painful.”

##### Patient-reported outcome measures (PROMs)

During the screening visit, subjects will be asked to complete a set of PROMs assessing their psychological traits. During each study period, subjects will be asked to complete an additional set of PROMs assessing their expectations, tiredness, and anxiety. The PROMs will be collected via questionnaires in the local language. Scores calculated from these questionnaires will be entered in the source paper documentation before entering into the electronic CRF. The subjects will be instructed on how to fill out the questionnaires.

The following PROMs will be completed during the screening visit, in the following order: (1) *Self-assessment of general health using the PROMIS Global-10 questionnaire*, a global health assessment tool that allows measurements of symptoms, functioning, and healthcare-related quality of life for a wide variety of chronic diseases and conditions; (2) *Self-assessment of self-efficacy using 3 items of the General Self Efficacy Scale (GSE)* [29], a psychometric scale that is designed to assess optimistic self-beliefs to cope with a variety of difficult demands in life; (3) *Self-assessment of trait anxiety using the General Anxiety Disorder-7 questionnaire (GAD-7)*, a brief scale on anxiety; (4) *Self-assessment of depression using the Patient Health Questionnaire-9 (PHQ-9)*, a diagnostic tool for mental health disorders, specific to depression; (5) *Self-assessment of pain catastrophizing using the Pain Catastrophizing Scale (PCS)* [30], a 13-item scale, broken into three subscales being magnification, rumination, and helplessness; (6) *Self-assessment of pain sensitivity using the Pain Sensitivity Questionnaire (PSQ)* [31].

The following PROMs assessing subject expectations will be completed at the beginning of each study period: (1) *Anxiety* will be assessed by asking subjects: “On a scale of 0–100, please rate, how anxious you are about the upcoming experiment, with 0 being ‘not anxious at all’ and 100 being ‘extremely anxious’”; (2) *Pain expectation* will be assessed by asking subjects: “On a scale of 0-100, please rate how much pain do you anticipate experiencing during the upcoming experiment, with 0 being ‘no pain at all’ and 100 being ‘pain as bad as you can imagine’”; (3) *Expectation of IMP-induced pain relief* will be assessed by asking subjects: “On a scale from 0 to 100, please rate how much pain relief you expect from the medication, with 0 being ‘expecting no relief’ and 100 being ‘expecting complete relief’.”

The PROMs assessing tiredness and anxiety will be assessed 5 h after IMP administration: (1) *Self-assessment of tiredness*. The subject will be asked: “On a scale of 0–100, please rate, how alert or sleepy you feel right now, with 0 being ‘very alert’ and 100 being ‘very sleepy’”; (2) *Self-assessment of state anxiety using the State and Trait Anxiety Inventory (STAI-Y)* [32].

## Hyperalgesia testing

The hyperalgesia testing is the same as described in the protocol for our related trial [IMI2-PainCare-BioPain-RCT3] see [2] for more details. Briefly, the intensity of the sensation elicited by calibrated mechanical pinprick stimuli will be assessed at the left (HFS-sensitized) and right (non-sensitized) sides. At the sensitized sites, the extent of the area of secondary hyperalgesia will be measured by locating the position at which subjects report a change in the intensity of the sensation elicited by mechanical pinprick stimuli delivered along 8 radial directions. The same approach will be used to assess the intensity and area of dynamic allodynia (if present), using a standardized Q-tip to deliver tactile stimuli.

### Plans to promote participant retention and complete follow-up {18b}

The plans to promote participant retention and complete follow-up are not the same as descibed in the protocol for our related trial [IMI2-PainCare-BioPain-RCT3] see [2] for more details.

### Data management {19}

The data management is the same as described in the protocol for our related trial [IMI2-PainCare-BioPain-RCT3] see [2] for more details. Briefly, data management will be performed by the Heidelberg University Computing Centre. Documentation of the responsibilities and delegation thereof will be maintained in the trial master file.

All aspects of the data management process have been specified in RCT3 [2] and are detailed in the Data Management Plan.

All source data arising from the trial will be kept by the investigator, who will provide direct access for trial-related monitoring, audits, ethics committee review, and regulatory inspection.

Case report forms for each subject will be provided to the investigator in electronic format and will serve to create the local source documentation in paper format. The investigator and delegated personnel will use these paper-form documents to record the source data information required by the protocol. The source data documentation will be entered into a validated electronic CRF system via a secured access to the Research Electronic Data Capture (REDCap) database hosted at the Heidelberg University computing center. The collected data will reside on secure servers of the Heidelberg University. Entry, corrections, and alterations of data within the system can only be performed by the investigator or other authorized personnel under their supervision and will be captured by the system’s audit trail. Users will be trained and receive access rights according to their role in the trial. All users will have access to the system and be able to review their data on an ongoing basis. After completion of the subject’s CRF, the CRF will be signed electronically by the investigator to confirm that the data are checked, complete, accurate, and in alignment with the source data. With database lock, the edit rights to the CRFs will be removed, but the investigator will retain access to view the CRFs. The nature and location of all source data/clinical documentation will be identified and documented by the investigator to ensure that all sources of original data required to complete the CRF are known to the sponsor and/or trial site personnel and are accessible for verification during trial-related monitoring, audits, relevant IEC review, and inspection(s). During trial conduct, the Heidelberg University Computing Centre is responsible for data security related to the data captured in the CRF.

Pharmacodynamic data will also be transferred to the Heidelberg database and data will be extracted centrally at Sapienza University where the data will be stored on secured servers. Derived data will be transferred to the relational database hosted at the Heidelberg University computing center. Pharmacokinetic concentrations and pharmacokinetic parameters measured by Welab will be uploaded by Welab to the relational database at the Heidelberg University computing center. Investigator site file and trial master files will be kept according to GCP.

### Confidentiality {27}

Subject trial data will be stored in a manner maintaining confidentiality in accordance with applicable regulatory requirements.

The source data will be pseudoanonymized and encoded (i.e., name and social security number), so to avoid the possibility to identify the individual persons.

### Plans for collection, laboratory evaluation, and storage of biological specimens for genetic or molecular analysis in this trial/future use {33}

This is described in detail for RCT3 [2] and is summarized here.

The analysis of drug levels at all the different sampling times will be performed on pharmacokinetic samples collected from trial subjects who will be randomized to receive an active treatment (tapentadol, pregabalin, or lacosamide). Placebo samples will be analyzed at a single time point around *t*_max_ of drugs.

Drug concentrations of tapentadol, pregabalin, and lacosamide will be analyzed by Welab Bioanalysis and ADME Development Department using a validated method under GLP (Good Laboratory Practice). The three drugs will be identified and quantified using HPLC method with tandem mass spectrometric detection (LC-MS/MS). Full details of the different analytical methods used will be described in the respective bioanalytical method validation report, and drug levels quantified in human plasma samples will be also reported in the respective bioanalytical report.

The bioanalytical laboratory will be unblinded in order to analyze multiple time points from the specific active treatments and single time points from corresponding placebos. The analysis will be conducted when the clinical study is finished but the dataset is not yet locked ensuring that sponsor remain blinded by recoding the subject’s numbers.

For each compound, relevant PK parameters will be calculated by standard non-compartmental methods for those subjects with sufficient plasma concentration data using Phoenix 64® WinNonLin® (Version 6.3 or later) with a log-linear terminal phase assumption. All reported sampling time deviations will be taken into consideration for evaluation of PK parameters.

For all three drugs, the following standard non-compartmental pharmacokinetic parameters and drug-exposure-related metrics will be estimated in each subject, including:

- *C*_max_: maximum plasma concentration.
- *t*_max_: time to reach maximum plasma concentration.
- λz: the terminal phase constant will be calculated by linear regression of the last phase of the curve (log concentration vs time).
- *t*_1/2_: terminal half-life will be determined with the expression *t*_1/2_= 0.693/λz.
- AUC0-t: Area under the plasma concentration-time curve from time zero to last quantifiable concentration calculated by the linear and/or log trapezoidal rule.
- AUC0-∞: The area under the curve of plasma levels vs time from zero to infinite will be obtained with the expression AUC0-∞= AUC0-t +Clast/λz, where Clast is the predicted plasma concentration at the last time measured.

A descriptive analysis will be provided for each derived PK parameter. Below limit of quantitation (BLQ) concentrations will be treated as zero for all statistical analyses.

Full details of the pharmacokinetic analysis and the corresponding statistical analysis of PK parameters will be described in the final report.

## Statistical methods

### Statistical methods for primary and secondary outcomes {20a}

**Fig. 3:**
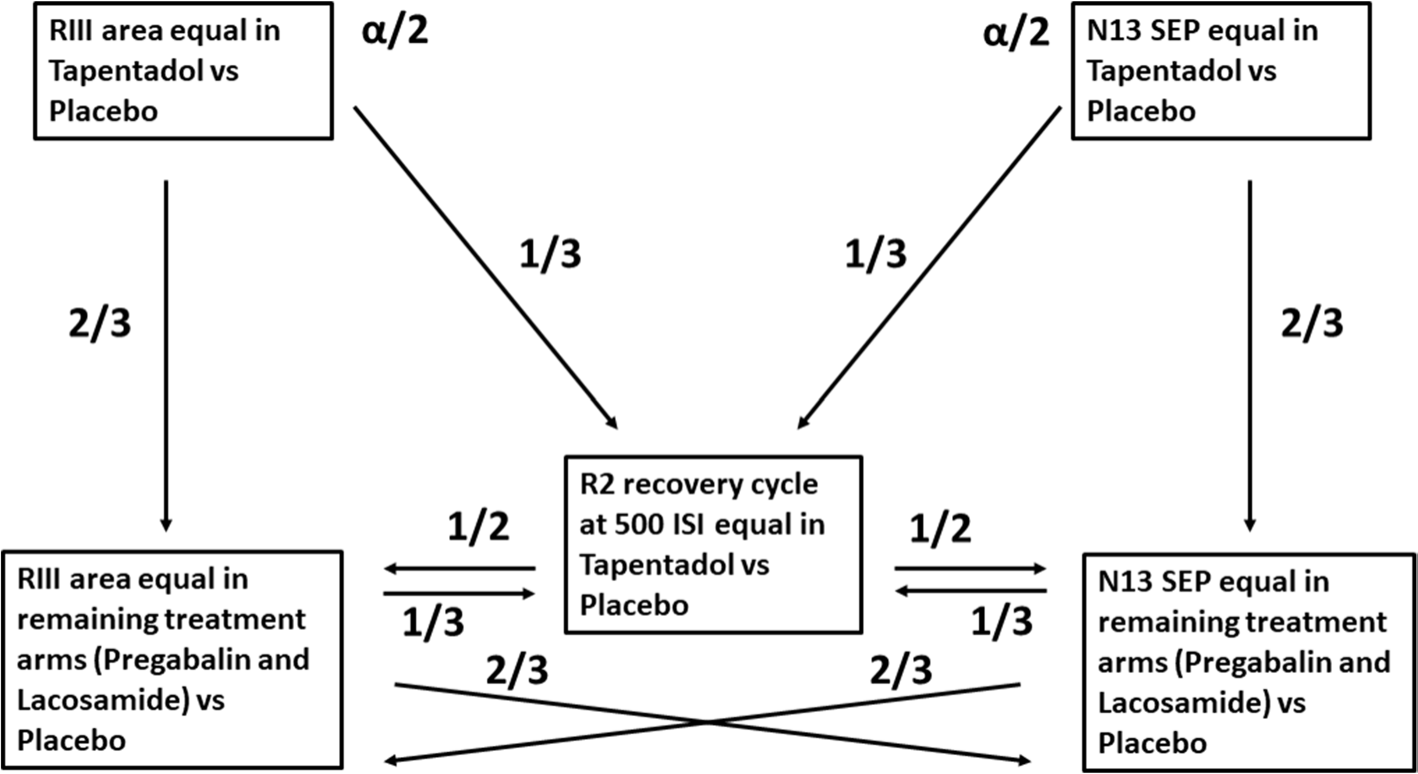
Statistical procedure

This and the following sections briefly specify the statistical analysis principles for the study. Final and detailed specifications on the quantitative analyses described here will be provided correspondingly in the statistical analysis plan to be finalized prior to unblinding of the study. The statistical procedure for the selected, confirmatory analyses will follow the sequentially rejective multiple testing approach described by Bretz et al. [33]. The two primary endpoints, change in the RIII area at the sensitized limb and in the N13-SEP amplitude after stimulation of the sensitized hand, will be tested for their differences between the treatment arm tapentadol versus placebo, first. This will be conducted in parallel, splitting the overall *α* equally between the endpoints’ tests, i.e., each test has a type I error of *α*/2. If any of these two tests shows significant differences, key secondary analyses will be conducted using the *α*-levels as passed on from initial/prior tests according to specified weights. The exact procedure (with the local levels as well as the weights with which to pass *α*-levels on) is illustrated by in Fig. 3.

#### Definition of the primary endpoints

RIII flexion reflex change endpoint data being repeated measurements across the four periods will be analyzed with a mixed effect model with treatment (4 levels), period (4 levels), and center and sequence as fixed effects. The variance-covariance structure for the repeated measure variable period should be chosen as UN (unstructured), TOEPH (heterogeneous Toeplitz), CSH (heterogeneous compound symmetry), ARH(1) (heterogeneous AR(1)), TOEP (Toeplitz), CS (compound symmetry), and AR(1) (first order autoregressive) in that order of preference.

The least squares (LS) mean difference of treatment active treatment versus placebo will be estimated based on this model and the estimate as well as the corresponding unadjusted confidence interval and *p*-value will be provided.

The analysis for the N13-SEP change is similar and details will be provided in the analysis plan.

If either (or both) of the primary endpoints’ tests show significant differences between tapentadol and placebo, in the sense that the *p*-value is below the corresponding local alpha level, the differences are confirmed and the testing procedure continues according to the graph above.

### Definition of secondary endpoints

These statistical analyses are considered confirmatory only provided they have received local *α*-levels (type I error greater than 0) from prior tests rejecting their corresponding null hypotheses. The method for testing the secondary endpoints comparing the other treatment groups versus placebo mirrors the model specifications of the primary analysis above but using the Dunnett-adjusted estimation and testing results to compare pregabalin and lacosamide with placebo respectively. The method for testing the key secondary endpoint for R2 recovery cycle is identical to the specifications of the primary analysis above.

### Interim analyses {21b}

No interim analysis is foreseen for this study.

### Methods for additional analyses (e.g., subgroup analyses) {20b}

The methods for additional analyses are the same as described in the protocol for our related trial [IMI2-PainCare-BioPain-RCT3] see [2] for more details. Briefly, several additional exploratory analyses will be done, such as variants of primary endpoint parameter extraction, additional functional biomarkers extracted from the recorded signals, item analyses of PROMs, pharmacokinetic-pharmacodynamic modelling, complex hierarchical modelling, estimation of variance, and effect size of candidate endpoints for future clinical trials. These are described in the statistical analysis plan.

The data collected in this trial, together with the data from the other three IMI2-PainCare-BioPain-RCTs and the preclinical studies of the BioPain project, will be subject to pharmacometric analyses with the intention to validate biomarkers that can translate from preclinical to clinical readouts [2]. As both drug concentrations and biomarker responses are measured at several time points post-drug administration, the relationship between drug levels and selected biomarkers will be explored and modelled. The pharmacometric analysis will be described in a separate pharmacometric analysis plan and will consist briefly in developing population pharmacokinetic/pharmacokinetic (PK/PD) models estimating the primary PK parameters (i.e., apparent volume of distribution, and total plasma clearance), the primary PD parameters (i.e., C50, the plasma or effect site concentration that elicits a response equal to half of the maximum attainable effect (EMAX), and their associated inter-individual variability.

Analyses of demographic data and other baseline characteristics will consist of descriptive summary statistics. While these tables may provide the summaries by groups (i.e., treatment sequences), there will be no statistical tests conducted on baseline characteristic differences. Data will also be listed. Details will be provided in the statistical analysis plan.

A reliability analysis will investigate the biomarker and their measurement characteristics using the following concepts, all based on the full analysis set. Detailed specifications will be provided in the statistical analysis plan. Assessment of the repeatability of a (individual) biomarker response measurement method will use the statistics described by Bland & Altman [34] (such as differences against means plots). Also, within and between subject variabilities will be estimated using the mixed AN(C)OVA models including the corresponding variance components according to the repeated measurements design. These include data across time points within a “visit” (e.g., during placebo treatment period) or across “visits” (e.g., baseline measurements across the treatments periods) as applicable. Should measurement methods of the same biomarker be compared the agreement statistics of Bland and Altman will be used. Assessment of the reproducibility will follow the statistics (such as difference limits, minimum significant difference, limits of agreement, etc.) as described in standard guidance documents. As applicable, further statistics presented for test-retest characteristics may include correlations (such as concordance correlation coefficients, intraclass correlation coefficients), coefficients of variations, and sensitivity to change statistics, e.g., smallest real difference.

Any changes to the planned statistical analyses, if considered prior to unblinding, will be described and justified in an amendment to the protocol (if study is still ongoing) and to the statistical analysis plan. Deviations from planned analyses will also be explained in the final study report.

### Methods in analysis to handle protocol non-adherence and any statistical methods to handle missing data {20c}

The methods in analysis to handle protocol non-adherence and any statistical methods to handle missing data are the same as described in the protocol for our related trial [IMI2-PainCare-BioPain-RCT3] see [2] for more details.

### Plans to give access to the full protocol, participant-level data, and statistical code {31c}

This will be determined at the end of the project period of IMI-PainCare.

## Oversight and monitoring

### Composition of the coordinating center and trial steering committee {5d}

The composition of the coordinating centre and trial steering committee are similar to the ones described in the protocol for our related trial [IMI2-PainCare-BioPain-RCT3] see [2] for more details. Briefly, this trial is part of the Biopain subtopic of the IMI2 PainCare project.

The PainCare Innovative Medicines Initiative (www.imi-paincare.eu) is a partnership between the European Union and the European pharmaceutical industry. IMI facilitates open collaboration in research to advance the development of and accelerate patient access to personalized medicines for the health and well-being of all, especially in areas of unmet medical need.

The overall coordination of the IMI-PainCare project is performed by the Project Coordinator who acts simultaneously as Co-Lead for the subproject BioPain including the IMI2-PainCare-BioPain RCTs (Prof. Rolf-Detlef Treede, Heidelberg University, Germany).

The RCT Lead will act as the international coordinating investigator who is responsible for the coordination of the Principal Investigators at multiple trial sites in multiple countries. The RCT Lead will be sponsor of the study (Prof. Andrea Truini, Sapienza University of Rome).

There will be one Principal Investigator at each trial site. If, at the trial site, the trial is conducted by a team of individuals, the investigator leading and responsible for the team is called the Principal Investigator, and the other individuals of the team are called investigators.

IMI PainCare project has been approved under the condition that the consortium carrying out the project implements an external ethics advisory board which (1) reviews the proper application of the relevant laws and guidelines containing ethical rules and the H2020 rules by the investigators; (2) provides advice to and monitors the activities of the investigators with regard to ethical issues; and (3) provides advice on the compliance with European ethical laws and regulations and with different guidelines, laws, and regulations of countries where studies are being performed. Practical implementation: ConsulTech, non-clinical partner of the study, will collect all relevant ethical documents and will ensure that all partners submit them on time. ConsulTech will then draw up a questionnaire for the ethics advisory board, which will allow it to check whether all important ethical requirements and documents have been submitted and that all legal guidelines have been adhered to.

### Composition of the data monitoring committee, its role and reporting structure {21a}

A data monitoring committee is not foreseen. Data management will be performed by the Heidelberg University Computing Centre. Documentation of the responsibilities and delegation thereof will be maintained in the trial master file. All aspects of the data management process are described in the Data Management Plan.

### Adverse event reporting and harms {22}

Adverse events will be documented from the time of enrollment up to the time of the last protocol scheduled contact. Subjects in the trial have the opportunity to report adverse events spontaneously. There will also be given a general incitement for events. Date of onset and termination for each adverse event, and impact of the test substance will be registered. The severity of the adverse event and the relationship between the treatment drug and the adverse event will be assessed as previously extensively described [2]. Investigators will report all serious adverse events to the sponsor as soon as possible and within 24 h, in order to report to the Competent Authority (CA) and the IEC. Handling of adverse events will follow GCP and applicable regulations as described previously [2].

### Frequency and plans for auditing trial conduct {23}

The frequency and plans for auditing trial conduct are similar to the ones described in the protocol for our related trial [IMI2-PainCare-BioPain-RCT3] see [2] for more details.

### Plans for communicating important protocol amendments to relevant parties (e.g., trial subjects, ethical committees) {25}

Any modifications to the protocol which may impact on the conduct of the study, potential benefit of the subject or may affect subject’s safety, including changes of study objectives, study design, subject population, sample sizes, study procedures, or significant administrative aspects will require a formal amendment to the protocol. Such amendment will be approved by the IEC and prior to implementation and notified to the health authorities in accordance with local regulations [2].

### Dissemination plans {31a}

A final report integrating trial results will be prepared. The Principal Investigator will provide the competent authority/ies and relevant IEC(s) with a summary of the trial results in accordance with applicable regulatory requirements.

The results of this trial will be publicly disclosed (EudraCT). The results (or parts thereof) of this trial will be published according to the Grant Agreement and Consortium Agreement of IMI-PainCare (grant No 777 500).

## Discussion

IMI2-PainCare-BioPain-RCT2 is one of four RCTs aiming at validating biomarkers of drug effects on nociceptive processing using the same trial design and IMPs. The design of IMI2-PainCare-BioPain-RCT3 on electrophysiological brain biomarkers has been published before [2]; two companion manuscripts describe IMI2-PainCare-BioPain-RCT1 (peripheral nerve excitability biomarkers) and IMI2-PainCare-BioPain-RCT4 (brain imaging biomarkers). By looking at spinal (flexor reflex RIII, somatosensory evoked potential N13) and brainstem biomarkers (blink reflex R2), RCT2 has the chance to profile the efficacy of the three model compounds (lacosamide, pregabalin, tapentadol) on mechanisms possibly contributing to brainstem and dorsal horn excitability in neuropathic pain patients, exploitable in preliminary pharmacological trials. The dorsal horn of the spinal cord is the first synaptic processing stage of the nociceptive system that is sensitive to activity-dependent modulation of synaptic strength and is under descending inhibitory and facilitatory control from the brainstem. Hence it is an attractive target for modulation by analgesic drugs.

The RIII component of the flexor reflex is elicited by electrical stimulation of nociceptive A-delta fibers. It is sensitive to altered signal processing in ventral horn, dorsal horn, and their descending controls from the brainstem. The N13 component of the somatosensory evoked potential is elicited by electrical stimulation of tactile Aß fibers that converge with nociceptive fibers on dorsal horn wide-dynamic range nociceptive neurons. It is sensitive to altered dorsal horn signal processing and its modulation by descending controls. The R2 component of the blink reflex is also elicited by electrical stimulation of tactile Aß fibers, but the signal pathways that determine its recovery cycle include brainstem reticular formation that also processes nociceptive inputs.

According to the sequentially rejective multiple testing design, two primary endpoints will be tested simultaneously at alpha/2; these are pairwise comparisons of one medication vs. placebo for one post-medication time point and one readout variable that are most likely to be significant, based on published literature (see power calculation). Three key secondary endpoints are assessed, given that alpha levels were passed successfully from one or both of the primary endpoints or from one or both of the other key secondary endpoints. All efficacy analyses will be done on data pooled across participating centers; efficiency of this multicenter study approach in human volunteers will be assessed by comparing across-center vs. within-center variability of the various readout variables.

In addition to the analysis of the individual RCTs, biomarker results and pharmacokinetic and pharmacodynamics analyses will be compared across trials and to preclinical studies and will possibly try to establish a latent variable model of underlying mechanisms at peripheral, spinal, and brain levels. Pain ratings will be included as another readout variable, and questionnaires on psychological traits predictors (e.g., catastrophizing, anxiety) will also be analyzed as potential predictors of pain and analgesia. By identifying specific mechanisms within different compartments of the nociceptive system in healthy volunteers, these same quantitative neurophysiological biomarkers, if well validated against clinically meaningful outcomes in patients, may be used for patient stratification and enrichment in later clinical trials, as encouraged by the recent EMA/CHMP/970057/2011 Guideline. This will accelerate the development of novel analgesics in several ways: preclinical prediction will be improved by using translatable readouts across species; clinical Phase 1 trials will benefit from biomarkers of target engagement and from human surrogate models predictive of clinical efficacy; clinical Phase 2 and 3 studies will benefit from tools for patient stratification.

## Trial status

This manuscript is based on protocol version 4.0 dated 12/06/2019. Recruitment started in Spring 2021 and is expected to be completed in March 2022.

## Data Availability

Access to data has been extensively described in the Data management {19} section. The trial protocol is available on request. An article containing the most important outcomes will be submitted for publication in a peer-reviewed journal after availability of the required dataset.

## Abbreviations

AE: Adverse event
AV: Atrioventricular
AR: Adverse reaction
BID: Bis in die: twice daily
CA: Competent Authority
*C*_max_: Peak concentration
CNS: Central nervous system
CRF: Case report form
IEC: Independent Ethics Committee
ECG: Electrocardiography or electrocardiogram
EFPIA: European Federation of Pharmaceutical Industry Associations
EMA: European Medicines Agency
EU: European Union
GCP: Good Clinical Practice
ICH: International Conference on Harmonization
IMI: Innovative Medicines Initiative (of the EU)
IMP: Investigational medicinal product (see “Definition of terms”)
LEP: Laser evoked potentials
K2-EDTA: Dipotassium ethylenediaminetetraacetic acid
*N*: Number (of subjects)
N13: Negative cervical component of SEP
PD: Pharmacodynamics
PI: Principal Investigator
PK: Pharmacokinetics
PK-PD: Pharmacokinetics-pharmacodynamics
PR: Interval of the ECG
PROM: Patient-reported outcome measure
R2: Late component of the blink reflex
RIII: Nociceptive flexion reflex
RCT: Randomized controlled trial
SAE: Serious adverse event
SD: Standard deviation
SmPC: Summary of Product Characteristics
SEP: Somatosensory evoked potentials
*t*_1/2_: Half-life
*t*_max_: Time to reach peak concentration

## Footnotes

1 EudraCT 2019-000942-36; https://clinicaltrialsregister.eu/ctr-search/trial/2019-000942-36/DK

2 EudraCT 2019-000755-14; https://clinicaltrialsregister.eu/ctr-search/trial/2019-000755-14/IT

3 EudraCT 2019-001204-37; https://clinicaltrialsregister.eu/ctr-search/trial/2019-001204-37/BE

4 EudraCT 2019-000908-15; https://clinicaltrialsregister.eu/ctr-search/trial/2019-000908-15/DK
